# Editorial for the Topic: MEMS in Italy

**DOI:** 10.3390/mi15121528

**Published:** 2024-12-23

**Authors:** Alberto Corigliano

**Affiliations:** Department of Civil and Environmental Engineering, Politecnico di Milano, 20133 Milano, Italy; alberto.corigliano@polimi.it

Microsystems or microelectromechanical systems (MEMSs) over the last thirty years have seen impressive development in terms of potentialities and diffusion [1]; they are now widespread as microsensors and/or micro-actuators and can be found in many objects of common use.

After an initial period in which MEMS development was mainly driven by the design and production of inertial sensors, i.e., accelerometers and gyroscopes, the MEMS world has more recently been enriched by many other devices, like piezo-actuated MEMSs. These innovations have been enabled by the introduction of new materials in micro-fabrication technologies and by the dramatic improvement of clean-room technologies.

Italy has become an important player in the MEMS industry in terms of its contributions to research and development, design, production and innovative applications.

The 22 articles collected in here cover a wide variety of scientific problems and solutions offered by the MEMS world, which is witnessing an impressive diversity of applications.

We can group the contributions of the topic into the following three main areas—materials and fabrication processes, innovative devices and innovative applications.

In the first area, new materials have been proposed [contribution 1], fabrication steps have been analyzed in depth [contribution 2], reliability issues have been discussed [contribution 3], the diffusion of additive manufacturing technologies for the creation of MEMSs has been critically described [contributions 4,5] and an overview of organic bioelectronics developments has been proposed [contribution 6].

Innovative devices have been proposed and analyzed, including piezo-micro-ultrasonic transducers [contribution 7], acoustic wave triggers [contribution 8], microcapsules [contribution 9], microgrippers [contribution 10], microphones [contribution 11], micromirrors [contribution 12] and gas sensors [contribution 13], confirming the great variety of devices that are now being proposed and that most probably will be transformed into future commercial products.

The applications mentioned in this topic concern gas storage [contribution 14], wearable devices [contribution 15] and drug delivery systems [contributions 16,17] for the biomedical field, space industry, the monitoring of soil in agriculture [contribution 18], nanoparticle size assessments [contribution 19], structural health monitoring [contribution 20] and communication systems [contributions 21,22].

The articles mentioned in this Editorial collectively represent a meaningful portion of research activities on MEMSs in Italy. They demonstrate significant advancements in technology for MEMS production and highlight the wide range of applications of these technologies in many fields, which span from the consumer market to agriculture and biomedical applications. The insights and innovations presented in this collection may offer new solutions to both long-standing and emerging challenges related to the underlying science of MEMSs, as well as their engineering and fabrication.
